# A diagnostic dilemma in pediatric proptosis—from suspected malignancy to benign orbital cavernous venous malformation: a case report

**DOI:** 10.1186/s13256-026-05889-0

**Published:** 2026-02-24

**Authors:** Mohd-Asyraaf Abdul-Kadir, Muhammad Adri Mohamed Shafit, Adzura Salam, Akmal Haliza Zamli

**Affiliations:** 1 Department of Ophthalmology, Kulliyyah of Medicine, International Islamic University Malaysia, Kuantan, Pahang Malaysia; 2 Department of Ophthalmology, Faculty of Medicine and Health Sciences, Universiti Malaysia Sarawak (UNIMAS), Kota Samarahan, Malaysia; 3 Department of Ophthalmology, Faculty of Medicine, Universiti Sultan Zainal Abidin (UniSZA), Kampung Gong Badak, Terengganu, Malaysia; 4 Department of Ophthalmology, Hospital Tengku Ampuan Afzan, Kuantan, Pahang Malaysia

**Keywords:** Proptosis, Case report, Cavernous venous malformation

## Abstract

**Background:**

Rapidly progressive proptosis in children can be caused by multiple causes. Distinguishing between these is challenging, especially when presentations mimic malignancy.

**Case presentation:**

A healthy Malay 3-year-old boy presented with a 1-week history of painless, progressive left eye proptosis. Initial imaging showed intraconal and extraconal orbital masses with prominent vascular channels, suggesting a vascular lesion. Two weeks later, he developed fever, lethargy, and worsening proptosis. Repeat imaging revealed lesion enlargement with intralesional hemorrhage, displacement of orbital structures, and a dilated superior ophthalmic vein. Differential diagnoses included rhabdomyosarcoma, orbital cellulitis, and venolymphatic malformation. Anterior orbitotomy and debulking were performed, and histopathology confirmed cavernous venous malformations with thrombosed vessels. At 6 months, there was no recurrence, but dense corneal scarring left the visual prognosis uncertain.

**Conclusions:**

Cavernous venous malformation is rare in children and usually progresses slowly. Acute enlargement from intralesional hemorrhage can mimic malignant or infectious disease. In pediatric orbital masses, careful clinical assessment, targeted imaging, and histopathological confirmation remain essential. Early multidisciplinary intervention is key to preserving vision when rapid progression threatens the eye.

## Introduction

Diagnosing pediatric orbital proptosis is inherently challenging due to its diverse etiologies. The rapid progression in our case, without preceding trauma, initially raised strong suspicion for malignancy. Differential diagnoses include neoplastic, infectious, and inflammatory causes, with orbital cellulitis being a common but often misleading presentation that can mimic rhabdomyosarcoma (RMS)—the most common primary orbital malignancy in children [1].

## Case Presentation

**Fig. 1:**
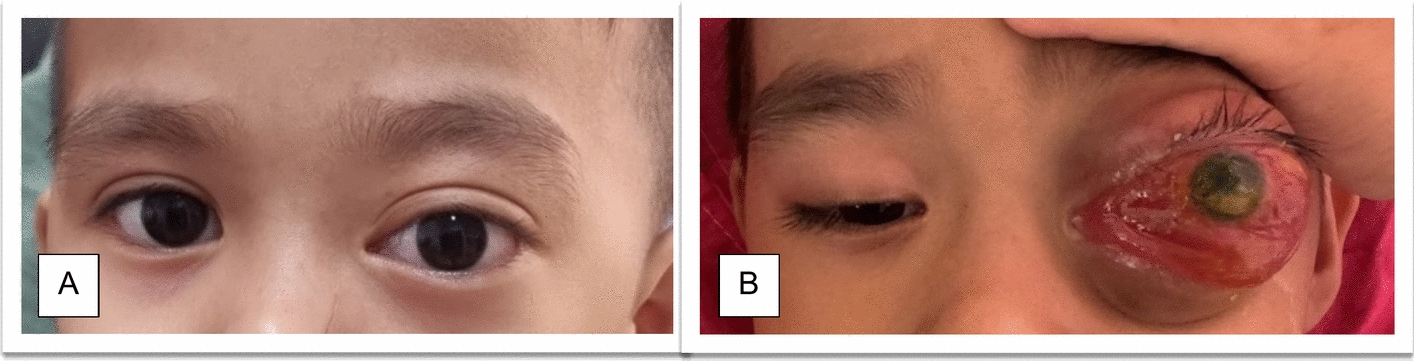
**A** The first presentation. Left eye notably appeared bigger and bulging. **B** Two weeks later, there was severe conjunctival chemosis and erythema, with severe proptosis, and the cornea appeared hazy

A previously healthy Malay 3-year-old boy presented with a 1-week history of progressive left eye proptosis to a district hospital on the east coast of Malaysia (Fig. 1A). He denied pain, redness, or discharge. Visual acuity was 6/12 in the right eye and 6/15 in the left. Pupils were reactive and symmetrical, with no leukocoria. There was a mild limitation in left eye abduction; other extraocular movements were intact. Hertel exophthalmometer showed a 7 mm difference in proptosis of the left eye compared with the right eye. The left eye was non-pulsatile, with white conjunctiva while posterior segment findings were normal. The right eye examination was unremarkable.

Perinatal history was uneventful. The child was born at term (38 weeks) from a non-consanguineous marriage. There was no relevant family history of orbital or systemic malignancy. There was no history of trauma preceded the symptoms.

Contrast-enhanced computed tomography (CECT) of the brain and orbits revealed both intraconal (0.8 × 1.1 × 1.2 cm) and medial extraconal (1.7 × 2.8 × 2.4 cm) masses, with the latter displacing the left medial rectus muscle laterally. Posteriorly, dilated tubular vessels demonstrated vivid contrast enhancement. The radiological impression was of a vascular tumor. The patient was initially referred for urgent tertiary evaluation on the following day; however, presentation to a tertiary center occurred only after a 2-week delay. This postponement was partly attributable to the family’s preference for traditional and complementary therapies prior to seeking definitive medical care.

He then exhibited new symptoms: fever, lethargy, reduced appetite, worsening left eye swelling, and watery discharge (Fig. 1B). Septic workup showed an elevated white cell count (21 × 10^9^/L) and a chest X-ray indicative of bronchopneumonia. Differential diagnoses included rhabdomyosarcoma (RMS), orbital cellulitis, and venolymphatic malformation (VLM) exacerbated by systemic illness.

**Fig. 2:**
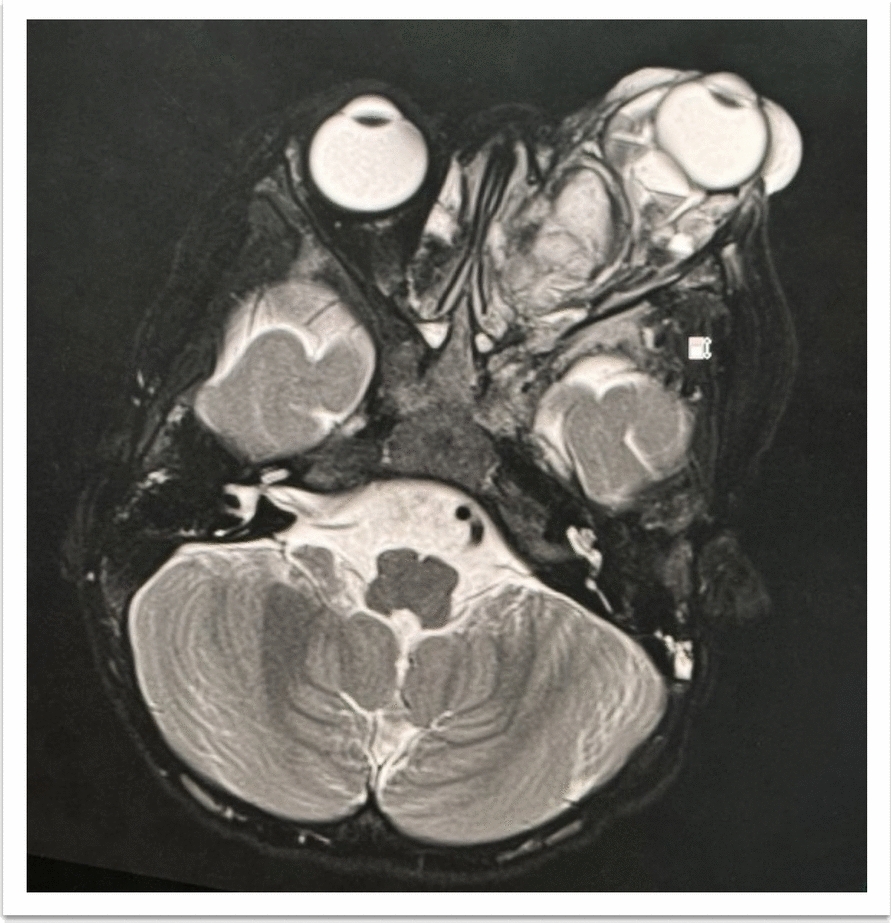
Magnetic resonance imaging showed left extraconal and intraconal orbital masses with fluid levels consisting of blood products with abnormal vessels and dilated superior ophthalmic vein

Repeat CECT showed increased lesion size with intraconal extension toward the left cavernous sinus, which was dilated. The mass displaced the superior ophthalmic vein (SOV) and compressed the lamina papyracea and maxillary sinus. Magnetic resonance imaging (MRI) confirmed the extraconal mass contained blood products and showed a tubular structure posteriorly, continuous with the dilated SOV (Fig. 2). The lobulated intraconal lesion, located medial to the optic nerve, contained fluid levels and blood products, without communication between the two masses and were heterogeneously hyperintense in T1, T2, and T2DF signals. Muscle and globe signals were normal. MRI of the brain did not demonstrate similar lesions. Imaging favored VLM or orbital lymphangioma.

**Fig. 3:**
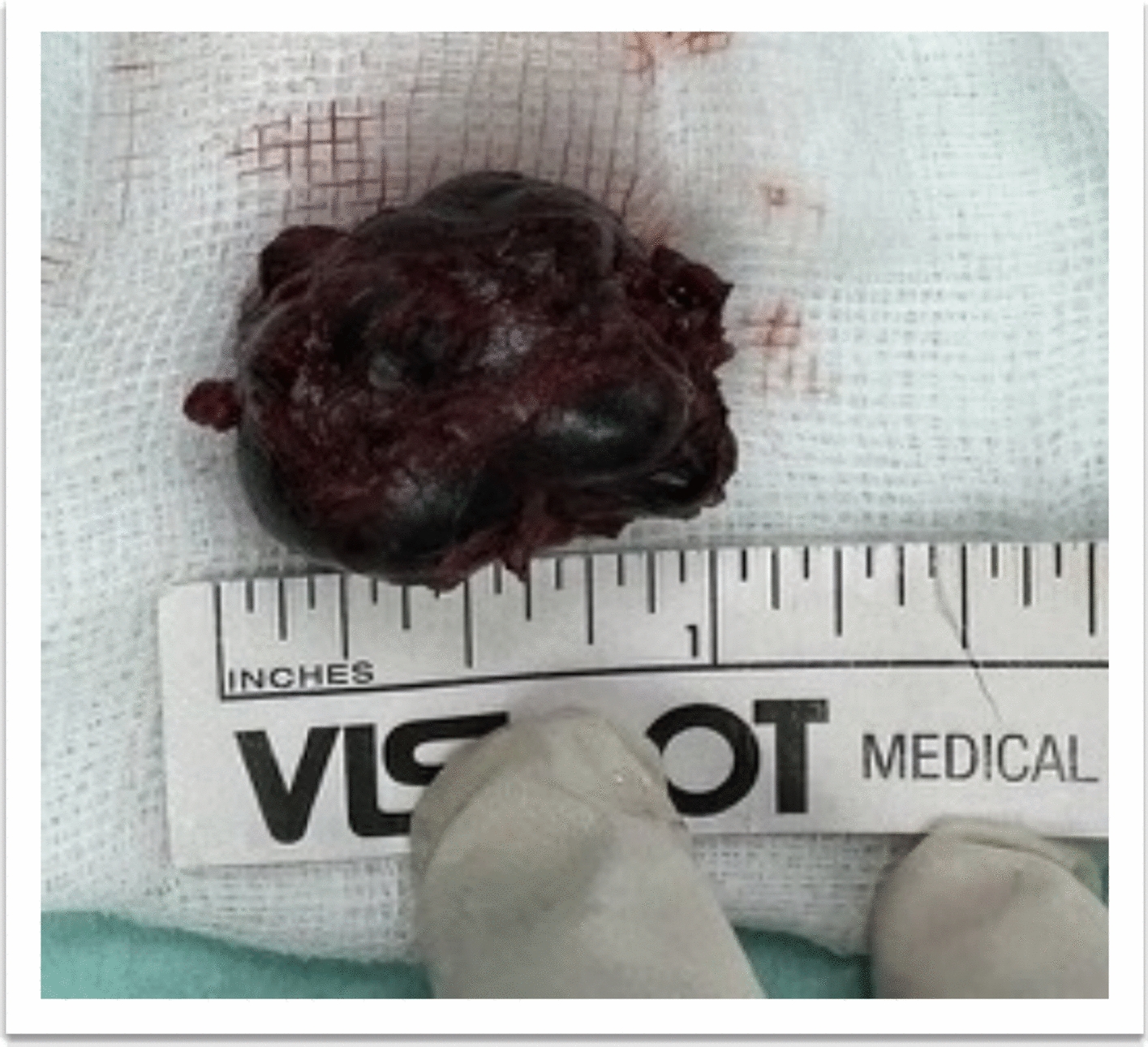
Well-defined mass following anterior orbitotomy and tumor debulking

After pneumonia treatment and stabilization, the patient underwent debulking and excision biopsy via a transconjunctival approach, due to worsening exposure keratopathy and concern for optic nerve compression. Histopathology confirmed a benign cavernous hemangioma with endothelial-lined vascular channels, thrombosed vessels, patchy D2-40 expression with absent atypia or malignancy (Fig. 3).

At 6-month follow-up, there was no clinical or radiologic evidence of lesion recurrence. Visual acuity in the affected eye was 6/60 and was limited by a dense central corneal scar secondary to prior exposure keratopathy, placing the patient at high risk of amblyopia. The child remains under close ophthalmologic surveillance with amblyopia therapy and has been referred for corneal assessment to optimize long-term visual potential.

## Discussion

Cavernous venous malformation (CVM) is rare in a 3 year-old child, and interestingly, the radiologic discordance findings were initially suggestive of VLM but were confirmed as CVM on histopathology. The acute worsening proptosis in this child has led to early worrying concern of malignant or more sinister causes.

The incidence of vasculogenic tumors in pediatric patients ranges from 5.5% to 22% [2]. In pediatric patients, capillary hemangioma is the most common vascular tumor in most studies; meanwhile, cavernous hemangioma or CVM is the most common vasculogenic lesion in adults and is rarely reported in children [3, 4]. The occurrence of orbital CVM in children support the theory of genetic hit to the vascular endothelium at the early stage of embryogenesis [5]. CVMs are low-flow venous malformations that present as solitary although few authors have reported multiple lesions within the orbit [6, 7]. Unlike neoplastic hemangioma, it is nonproliferative and encapsulated, with no tendency to hemorrhage [8, 9]. They typically follow an indolent course, with proptosis progressing slowly until the lesion reaches a size sufficient to produce functional deficits such as diplopia or optic neuropathy [10]. Acute and subacute presentation of orbital CVMs are uncommon, with most of the reported cases being in adults [11–13].

Imaging plays a pivotal role in the evaluation of orbital vascular lesion, demonstrating the locality, extension, and hemodynamic status, which provide a valuable guide in management strategies.

Classically, on computed tomography (CT) and MRI, CVMs appears as a solitary round, ovoid, and rarely lobulated well-defined mass with progressive contrast enhancement with time [15].

In contrast, our patient’s imaging further illustrates the diagnostic pitfalls. Well-circumscribed lesions with fluid levels suggestive of an intralesional hemorrhage had shifted clinical suspicion toward a benign vascular cause. Furthermore, the multiplicity and lesion enlargement on MRI with dilated superior ophthalmic vein and cavernous sinus raised concern regarding the hemodynamics of the lesion and possible connection with systemic venous system as seen commonly in distensible venous malformation and VLM [7]. The discordant radiologic features highlight an important limitation of imaging alone in pediatric orbital vascular lesion.

The intralesional hemorrhage explained the acute worsening of proptosis in our patient although the findings are more pathognomonic in VLM and rather uncommon in CVM. Several mechanisms accounted for this phenomenon, including underlying endothelial fragility within the abnormal vascular channels, hemodynamic stress in relation to systemic infection, or subclinical trauma. In addition, thrombosis within the lesion may have contributed to localized vascular congestion and rupture [10, 14, 15]. Furthermore, the venous stasis may induce hypercellularity and stromal changes [8]. Additionally, venous thrombosis within the CVM may mimic acute orbital syndrome, as it can provoke painful ophthalmoplegia [16]. These mechanisms highlight that, although rare, spontaneous intralesional hemorrhage in CVM should be considered when it enlarges rapidly and presents atypically in children. In our case, we concluded the slow hemodynamics of CVM with thrombosis and the systemic infection may have propagated the spontaneous intralesional bleeding that led to rapid progression of the disease.

Our patient underwent anterior orbitotomy via an extended trans-caruncular and inferior transconjunctival approach due to imminent visual risk, including optic neuropathy and severe exposure keratopathy. Histopathological confirmation was vital, highlighting that imaging alone may not suffice for definitive diagnosis of the case, management planning, and long-term prognostication for the child. Given the rarity of hemorrhagic presentation in CVM, the histopathological diagnosis was reviewed by two independent pathologists. Both confirmed the presence of cavernous hemangioma characterized by endothelial-lined vascular channels and thrombosed vessels, without features of malignancy or VLM.

The delay between urgent referral and tertiary evaluation contributed to disease progression and ocular surface complications. This interval is best interpreted as a healthcare access and health-seeking behavior issue rather than a cultural critique [17, 18]. Emphasizing the importance of evidence-based treatment may help foster parental confidence in modern healthcare and strengthen trust in medical decision-making.

## Conclusion

CVM is rare in young children and typically indolent. This case highlights that acute hemorrhagic enlargement of orbital CVM with thrombosis can mimic malignant or infectious orbital disease and is potentially vision-threatening. In evaluation of rapidly progressing pediatric orbital mass, clinicoradiological correlation alone may be insufficient, and histopathological confirmation remains essential to guide timely diagnosis and vision-preserving intervention.

## Data Availability

The materials are available from the corresponding author on reasonable request.
